# Immediate crossover fatigue after unilateral submaximal eccentric contractions of the knee flexors involves peripheral alterations and increased global perceived fatigue

**DOI:** 10.1371/journal.pone.0293417

**Published:** 2024-02-12

**Authors:** Jennifer Gioda, Flavio Da Silva, Florian Monjo, Baptiste Corcelle, Jonathan Bredin, Enzo Piponnier, Serge S. Colson

**Affiliations:** 1 LAMHESS, Université Côte d’Azur, Nice, France; 2 LIBM, Université Savoie Mont Blanc, Chambéry, France; 3 Centre de Santé Institut Rossetti-PEP06, Nice, France; University of Hartford College of Education Nursing and Health Professions, UNITED STATES

## Abstract

After a unilateral muscle exercise, the performance of the non-exercised contralateral limb muscle can be also impaired. This crossover fatigue phenomenon is still debated in the literature and very few studies have investigated the influence of eccentric contractions. This study was designed to assess neuromuscular adaptations involved in the crossover fatigue of the non-exercised contralateral knee flexor muscles. Seventeen healthy young men performed a unilateral submaximal eccentric exercise of the right knee flexors until a 20% reduction in maximal voluntary isometric contraction torque was attained in the exercised limb. Before (PRE), immediately after exercise cessation (POST) and 24 hours later (POST24), neuromuscular function and perceived muscle soreness were measured in both the exercised limb and non-exercised limb. In addition, global perceived fatigue was assessed at each measurement time. At POST, significant reductions in maximal voluntary isometric contraction were observed in the exercised limb (-28.1%, p < 0.001) and in the non-exercised limb (-8.5%, p < 0.05), evidencing crossover fatigue. At POST, voluntary activation decreased in the exercised limb only (-6.0%, p < 0.001), while electrically evoked potentiated doublet torque was impaired in both the exercised limb and the non-exercised limb (-11.6%, p = 0.001). In addition, global perceived fatigue significantly increased at POST (p < 0.001). At POST24, all measured variables returned to PRE values, except for perceived muscle soreness scores exhibiting greater values than PRE (p < 0.05). A possible cumulative interaction between peripheral alterations and global perceived fatigue may account for the immediate crossover fatigue observed in the non-exercised limb.

## Introduction

After a strenuous unilateral exercise, a decline in muscle force or power production of the exercised muscle and/or an increase in subjective sensations (e.g., perceived fatigue, perceived muscle soreness) associated with the task performed is commonly reported. This reduced muscle force production defined as performance fatigability [1] is commonly attributed to interactions between peripheral and central factors including impairments of contractile function distal to the neuromuscular junction and adjustments within the central nervous system at the spinal and/or the supraspinal level [2]. Interestingly, during maximal or fatiguing unilateral contractions, involuntary increased electromyographic activity and force production are observed in the non-exercised contralateral homologous muscles [3, 4]. In addition, after a fatiguing unilateral exercise, performance fatigability can occur in the non-exercised contralateral muscle, but this observation is still disputed in the literature [5].

Since the beginning of the millennium, numerous studies have sought to investigate the so-called “crossover” fatigue, defined here as the performance fatigability of a non-exercised contralateral homologous muscle following unilateral muscle exercise of the ipsilateral muscle [6]. Although evidenced many times [4, 7–11], some studies have failed [12, 13] to report crossover fatigue. A recent systematic review with meta-analysis [5] concluded that the conflicting literature neither supports the existence of crossover fatigue nor “non-local muscle fatigue” (i.e., fatigue occurring in any non-exercised heterologous muscle). The moderators that were examined (i.e., study design, age, sex, training status, homologous vs. heterologous muscles, upper body vs. lower body, type of outcome measure, time of measurement, fatigue severity) had trivial effects on crossover fatigue. However, this meta-analysis did not take the influence of the type of contraction (i.e., dynamic or isometric) into consideration. While most of the crossover fatigue studies implemented isometric contractions [4, 7, 10, 11, 14, 15], only four studies have used eccentric contractions, yet providing conflicting results [8, 9, 13, 16].

Eccentric contractions are known to generate performance fatigability of the exercised muscles that can be associated with contractile alterations [17–19], delayed muscle soreness and pain [8, 9], as well as central drive alterations [9, 17–19]. To date, after a unilateral eccentric exercise, increased [16], unchanged [13] or reduced force production [8, 9] is reported in the non-exercised contralateral homologous muscle. Due to the variability of the study designs, it is extremely challenging to explain the discrepancies observed among these studies. Interestingly enough, however, when considering the two studies that have evidenced crossover fatigue [8, 9], an interaction between central factors (i.e., reduced voluntary activation or muscle activity) and subjective sensations (i.e., increased perceived pain) seemed to contribute to the performance fatigability observed in the contralateral homologous muscle.

In crossover fatigue studies, the performance fatigability of the contralateral homologous muscle is generally assumed to encompass complex fatigue-induced central adjustments [20, 21] possibly leading to reduced central drive to the non-exercised muscle [7, 9–11]. In addition, peripheral factors involving the dispersion of metabolites through the cardiovascular system and/or the presence of heat shock proteins in resting muscles have been assumed to account for the decreased performance of the non-exercised contralateral muscle [5, 20]. As far as we know, only one study reported an impairment of the resting electrically evoked twitch associated with reduced performance of the contralateral first dorsal interosseous muscle [4]. Finally, subjective sensations such as pain, effort or increased perception of fatigue could also contribute to the altered performance of the contralateral homologous muscle [5, 8, 9, 20], even though such measures were scarcely performed in crossover fatigue studies [8]. Consequently, even if the underlying mechanisms accounting for crossover fatigue are still debated, it has been proposed in a recent meta-analysis that a complex interaction between peripheral and central factors associated with increased subjective sensations is likely to occur [5]. This suggestion is in accordance with some of the results reported in the two crossover fatigue studies using unilateral eccentric exercise [8, 9], although these two studies were not included in this meta-analysis [5].

To date, most of crossover fatigue studies were performed in the knee extensors or in the elbow flexors and the occurrence of crossover fatigue in the knee flexors is still unknown. In addition, recent studies investigating the etiology of neuromuscular fatigue of the knee flexors reported: i) conflicting results and, ii) differences in neuromuscular fatigue etiology between knee extensors and flexors [22, 23]. More importantly, it has been suggested that there is a need to better understand knee flexor muscles fatigue [23]. In this context, this study investigated whether a unilateral submaximal eccentric exercise of the knee flexors could induce a crossover fatigue in the non-exercised contralateral homologous muscle. Concomitant assessment of neuromuscular function with measures of perceived fatigue and muscle soreness was intended to ascertain the influence of both peripheral and central factors and subjective sensations in crossover fatigue. Based on recent studies [8, 9], we hypothesized that the submaximal eccentric fatiguing exercise would induce crossover fatigue, thus involving a cumulative central activation failure, peripheral alterations in both the exercised and non-exercised muscles and increases in perceived fatigue and muscle soreness. We also scrutinized the possible influence of involuntary increased electromyographic activity of the contralateral non-exercised muscles during the exercise of the ipsilateral muscle on crossover fatigue. Finally, due to the well-acknowledged long-lasting effect of eccentric exercises on performance fatigability [8, 9], the performance of both the exercised and contralateral non exercised muscles were assessed 24 hours after the exercise.

## Methods

### Participants

Based on a recent study reporting crossover fatigue after eccentric exercise [9], G*Power (version 3.1.9.4; Kiel University, Kiel, Germany) was used to estimate sample size a priori. The sample size determination led to the participation of ten individuals in a repeated-measure, within-between interaction analyses with a power set at 0.95. Seventeen healthy young men [mean ± standard deviation (SD); age: 23.8 ± 4.6; body mass: 77.2 ± 10.2 kg; height: 180.5 ± 3.9 cm] recruited between March and September 2021 took part in this experimental study. A sensitivity power analysis (α = 0.05, power = 0.95) led to a large effect of f = 0.47 for the sample size of the participants included in this study. In order to be part of the study, participants had to be involved in regular recreational sport activities, free of any medical contraindication to physical activity. Neither could they have experienced musculoskeletal, neurological, or orthopedic disorder in the lower limbs for at least six months. All volunteers were fully informed about all risks, discomforts, benefits of the study and each participant signed a written informed consent form before the beginning of the experiments. Individual anonymous data were collected by the investigators throughout the experimental procedures that were conducted in accordance with the latest version of the Declaration of Helsinki (2013). Approval was obtained from the Protection Committee of People for Biomedical Research Southeast III, France (Authorization Number 2020-A02811-38).

### Experimental procedure

**Fig 1:**
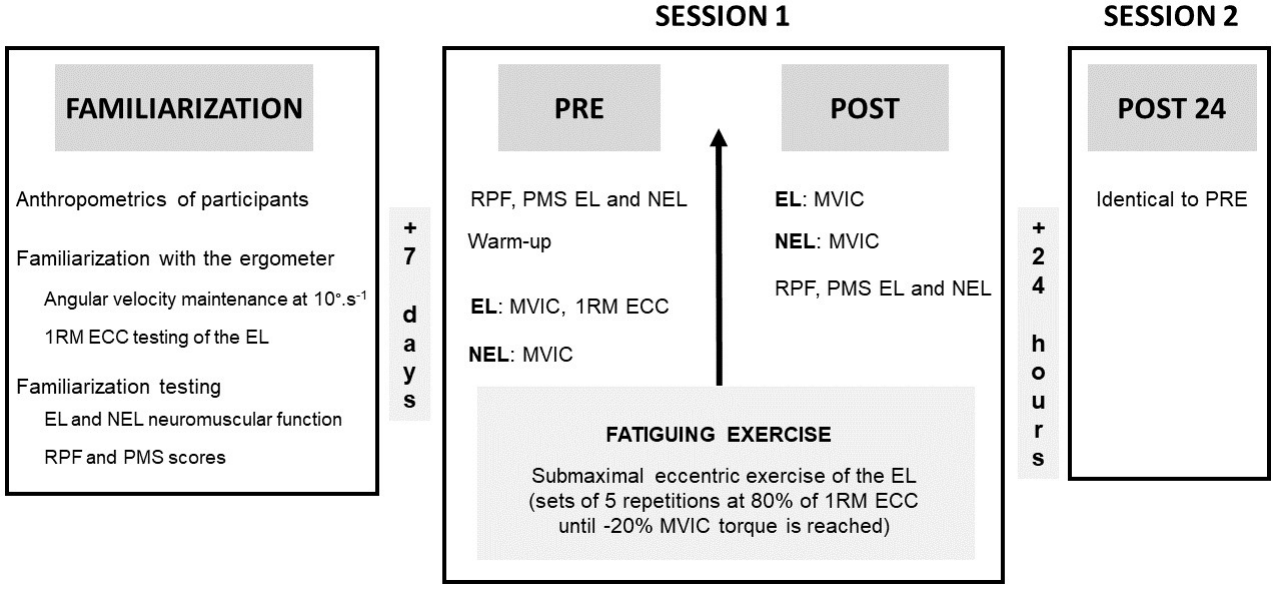
Overview of the experimental procedure. After a familiarization session, a first session (Session 1) assessed the neuromuscular function of both the EL (exercised limb) and the NEL (non-exercised limb), as well as global perceived fatigue (RPF) and perceived muscle soreness (PMS), before (PRE) and immediately after (POST) a unilateral submaximal fatiguing eccentric exercise of the EL knee flexors. A second session (Session 2) performed 24 hours after the exercise (POST24) was similar to PRE testing. Maximal voluntary isometric contraction (MVIC), 1 Repetition Maximum eccentric contraction (1RM ECC).

The participants were required to attend the laboratory in three occasions (Fig 1): i) a familiarization session, ii) a testing session including different measurements realized before (PRE) and immediately after (POST) the unilateral fatiguing exercise performed on the right lower limb (Session 1), and iii) a testing session performed 24 hours after the fatiguing exercise (POST24; Session 2).

One week prior to the testing session, participants were acquainted with the equipment, the experimental procedures, and the neuromuscular function assessment (see details below) on both limbs. During the familiarization session, a particular focus was put on the angular velocity to be maintained (i.e., 10°.s^-1^ provided by real time feedback on a screen placed in front of the participants) during the unilateral eccentric contractions of the right exercised limb (EL). Once fully familiarized with all the procedures on both limbs, the 1 Repetition Maximum eccentric contraction (1RM ECC) of the EL was determined by adding loads until the participant could no longer control the angular velocity of 10°.s^-1^. Loads of 5kg were added during each trial according to the participant’s individual responses. When approaching the 1RM ECC, lighter loads of 2kg and/or 1kg were used. Rest periods of 90s were allowed between trials. This familiarization procedure allowed a rapid 1RM ECC determination during the testing session starting from 90% of 1RM ECC.

The two testing sessions (i.e., Session 1—PRE and Session 2—POST24) started with the measures of rate of perceived fatigue (RPF) and perceived muscle soreness (PMS) scores. A standardized warm-up including the “Extender” (i.e., the leg was maintained in an extended position for 5s followed by 5s to relax and reach again the extended position) and the “Diver” (i.e., the position was attained in 3s followed by 1s to return to the starting position) exercises [24] and a single-leg bridges (i.e., 1s to reach the extension position, held 2s and 1s to return to the starting position) exercise were performed on both limbs for each exercise (i.e., 2 sets of 6 repetitions on each lower limb; one limb was exercised after each other). This was followed by 10 submaximal isometric contractions of both limbs in the isometric testing position. The intensity (i.e., in the percentage of perceived maximal force production) of the submaximal contractions interspaced with 30s of rest was progressively increased (i.e., ~30%, 4 contractions; ~50%, 3 contractions; ~70%, 2 contractions and ~90%, 1 contraction). Then, neuromuscular function was assessed as follows: i) two maximal voluntary isometric contractions (MVIC) with a 100-Hz superimposed (Dt_100Hz_ Sup) paired electrical stimulation delivered over the MVIC plateau, followed by potentiated stimulations elicited at rest at 2 (100Hz paired stimulus), 4 (10Hz paired stimulus) and 6 s (1Hz single stimulus), and ii) the determination of the 1RM ECC of the EL. The EL was always tested first followed by the non-exercised limb (NEL) measures. For the measurements performed immediately after the unilateral fatiguing exercise (i.e., POST), only one MVIC with superimposed and potentiated stimulations was assessed on both the EL and NEL, followed by the measure of RPF and PMS scores.

### Unilateral fatiguing eccentric exercise

The fatiguing exercise involved repetitive sets of five unilateral eccentric contractions of the right EL knee flexors at 80% of the 1RM ECC measured in PRE. Contractions were performed at an angular velocity of 10°.s^-1^ (i.e., contraction duration: ~5s) starting from a position combining 65° of hip flexion and 90° of knee flexion to a ending position combining 40° of hip flexion and 30° of knee flexion (0° = full extension of the hip and the knee). After each contraction, participants were passively lifted into the starting position by experimenters, providing a 10-s rest period. A 25-s rest interspaced sets of submaximal eccentric contractions. A MVIC of the EL was performed at the end of each set to evaluate the level of force decrement. Sets were repeated until a 20% MVIC force decrement was reached on EL.

### Measurements

#### Knee flexors’ torque

A specific ergometer (Hamtech device, Human Kinematic, Carros, France) allowing reliable and reproducible measurements in isometric and dynamic conditions [25] was used to assess the unilateral force production of knee flexors of both lower limbs. Force of each lower limb was collected using force transducers (S-beam, LS02-s, Tech Co. Ltd, Shenzhen, China; capacity: 1000N) inserted into the frame of the ergometer. Each force transducer was connected to a lever arm that can moved vertically to adapt the positioning according to the participants’ anatomical characteristics. The contact point of this lever arm was positioned 5 cm above the external malleolus on the Achilles tendon. A Biopac MP 150 (Biopac Systems, Inc., Goleta, CA, USA) was used to record force production at a sampling frequency of 1kHz. Force was converted into torque during offline data processing using participants’ lever arms (i.e., distance between the lateral tibial condyle and contact point of the lever arm connected to the force transducer). The ergometer also included a potentiometer (P4500, Novotechnik U.S., Inc., Southborough, MA, USA) allowing measurements of angular velocity. To stabilize the participants’ pelvis during force production, two elastic bands were positioned ~5cm above participants’ sacroiliac joint and below the gluteal fold. Isometric contractions were measured on both lower limbs (EL and NEL) in a standardized position with the hip and the knee flexion angles set at 40° and 30°, respectively (0° = full extension). Dynamic eccentric contractions of the EL (i.e., 1 RM ECC and contractions performed during the unilateral fatiguing eccentric exercise) started from a position combining 65° of hip flexion and 90° of knee flexion to a final position combining 40° of hip flexion and 30° of knee flexion.

#### Knee flexors’ surface electromyography

Surface electromyography of the biceps femoris (BF) muscles of both lower limbs was collected using pairs of surface electrodes (Ag-AgCl, diameter = 10mm; inter-electrode distance = 20mm; Contrôle-Graphique, Brie-Comte-Robert, France) placed in accordance with the SENIAM recommendations [26]. A reference electrode was placed on the lateral tibial condyle of the tested lower limb. Indelible ink marks ensured identical repositioning during the entire experiment. Low-resistance impedance (< 3kΩ) was obtained by shaving and slightly abrading the skin with emery paper. Electromyographic signals were recorded at a sampling frequency of 2kHz using the Biopac MP150 system (Biopac Systems, Inc., Goleta, CA, USA; bandwidth frequency = 10 – 500Hz, common mode rejection ratio = 110dB, Z Input = 1000MΩ, gain = 1000).

#### Knee flexors’ electrical stimulation

Percutaneous electrical myostimulation (400V and 1ms duration rectangular pulse) was delivered by an electrical stimulator (DS7, Digitimer Ltd., Hertfordshire, UK) through self-adhesive rectangular electrodes (5cm × 9cm—Stimex, Wetzlar, Germany) placed on the participants’ lower limbs. The cathode was positioned on the hamstrings’ proximal part (i.e., below the gluteal fold) and the anode was located at the popliteal fossa. Electrodes positions were marked on the skin to guarantee similar placement during the entire experimental procedure. The location of the stimulation electrodes was based on previous publications investigating neuromuscular function and neuromuscular fatigue of the knee flexors [23, 27, 28]. Maximal twitch force and maximal amplitude of the BF compound muscle action potential were determined at rest by progressive stimulation intensity increments on both lower limbs (i.e., starting at 10 mA, followed by 20 mA increments and adjusted with 5 mA changes when the twitch force plateaued). The stimulation intensity was further increased by 20% (EL: 142.1 ± 24.9 mA, NEL: 148.2 ± 31.0 mA) to warrant adequate assessment of knee flexors’ neuromuscular function.

#### Ratings of global perceived fatigue and muscle soreness

The notion of perceived fatigue, which was explained to the participants as “a feeling of diminishing capacity to cope with physical stressors” [29] was assessed using the French translated and validated version of the Rating-of-Fatigue Scale [30]. Ratings of perceived fatigue (RPF) accounting for participants’ global fatigue were scored from 0 to10 (0, not fatigued at all; 10, total fatigue and exhaustion–nothing left).

Perceived muscle soreness (PMS) was measured in both lower limbs using a visual analog scale. A steady 25-N pressure was applied with a 0.5cm diameter cylindrical object just above and below surface electromyography electrodes of the BF on both the EL and NEL [31]. Participants had to score their pain perception from 0 to10 (0, no pain; 10, worst pain).

### Data analysis

For PRE, POST and POST24 measurements, the highest MVIC peak torque value produced by both lower limbs was retained for data analysis. Root mean square (RMS) values of BF surface electromyography signals were calculated during a 500-ms period over MVIC. BF RMS was normalized to the respective BF compound muscle action potential (i.e., BF RMS/M) recorded at each time point. Potentiated torques evoked by electrical paired stimuli at 100 Hz (Dt_100Hz_) and 10 Hz (Dt_10Hz_) were used as peripheral fatigue indicators. The ratio Dt_10Hz_-to-Dt_100Hz_ was also computed to assess the possible occurrence of low-frequency fatigue [32]. Along with BF RMS/M, the maximal voluntary activation level (VA) and the central activation ratio (CAR) of the knee flexors were calculated and served as central fatigue indicators [33, 34]. VA and CAR were computed according to the following formulas:

$$VA\left(\%\right)=\left(1-\left(\frac{{Dt}_{100Hz}\;Sup}{{Dt}_{100Hz}}\right)\right)\times 100$$

$$CAR\left(\%\right)=\left(\frac{Voluntary\;torque\;before\;{Dt}_{100Hz}Sup}{Voluntary\;torque\;before\;{Dt}_{100Hz}Sup+\;{Dt}_{100Hz}Sup}\right)\times \;100$$

During the unilateral fatiguing eccentric exercise, the number of contractions performed and the amount of total work calculated using torque-time integral (i.e., area under the torque-time curve) were collected. The amount of torque-time integral produced for each submaximal fatiguing eccentric contraction during each seat was computed. According to the number of sets performed by each participant, the individual total duration of the submaximal fatiguing exercise was divided in four consecutive periods, each representing 25% of the total duration of the exercise. The MVICs performed at the end of each were not included in the torque-time integral calculation. These MVICs were expressed as a percentage of the initial MVIC value produced at PRE and were linearly interpolated between the nearest values at 25%, 50% and 75% of the individual total duration in order to describe the evolution of the participants’ MVIC throughout the entire duration of the submaximal fatiguing exercise. Similarly, the BF RMS of both the EL and NEL measured during each eccentric contraction of the submaximal fatiguing exercise (computed over the entire 5s duration of the contraction) and expressed as a percentage of the initial value measured at PRE were linearly interpolated between the nearest values at 25%, 50% and 75% of individual total duration of the exercise. The MVIC and BF RMS values retained at 100% of the number of sets performed corresponded to the value measured after the last set of each participant.

### Statistical analyses

The normality of the distribution of each variable was tested using the Kolmogorov–Smirnov test. The influence of the unilateral fatiguing eccentric exercise on the different variables measured was studied using generalized linear mixed-effect models (GLMMs) with linear distribution for normally distributed data. For non-normal distribution, the data was adjusted to the best fitting model in the GLMM. Models were tested with and without inclusion of a random effect (i.e., participant) using the Akaike information criterion (AIC) and the model with the lowest score was retained [35]. The GLMMs comprised limb (EL and NEL) and time (PRE, POST and POST24) fixed effects for MVIC, VA, CAR, BF RMS/M values, BF compound muscle action potential values, Dt_100Hz_, Dt_10Hz_, Dt_10Hz_-to-Dt_100Hz_ ratio, and PMS scores. RPF scores were tested with a fixed effect of time only. Throughout the fatiguing exercise, the time course of MVIC and torque-time integral values was analyzed with a fixed effect of time whereas BF RMS were studied with fixed effects of limb (EL and NEL) and time (25%, 50%, 75% and 100% of the exercise duration). Sidak adjusted pairwise comparisons were used when a significant interaction or main effect was observed. Pearson correlation analyses were performed in both the EL and NEL to assess the relation between PRE-POST MVIC reduction and the immediate alteration of central and peripheral fatigue indicators (i.e., VA, CAR, BF RMS/M values, BF compound muscle action potential values, Dt_100Hz_, Dt_10Hz_) as well as with changes in RPF and PMS scores. To test the possible inter-limb relation, Pearson correlations were done between MVIC reduction in the EL and MVIC reduction in the NEL, as well as between EL and NEL peripheral alterations. Correlation coefficient values were interpreted as recently proposed [36]. Significance was set at p < 0.05. Statistical analyses were performed using IBM SPSS Statistics software (IBM Corp., version 25 for Windows, Armonk, NY, USA). Cohen’s *d* effect size (ES) were computed to determine the importance of significant results (i.e., 0.2 < small < 0.5; 0.5 ≤ medium < 0.8; large ≥ 0.8). Unless specified, data is expressed as mean ± SD (standard deviation) in the manuscript, in the table and figures.

## Results

### Unilateral eccentric fatiguing exercise

The 1RM ECC determined for all participants was 177.31 ± 40.15 N.m. The number of contractions performed and the total torque-time integral produced were 37.65 ± 14.59 and 54.21 ± 21.03 kN.m.s, respectively.

**Fig 2:**
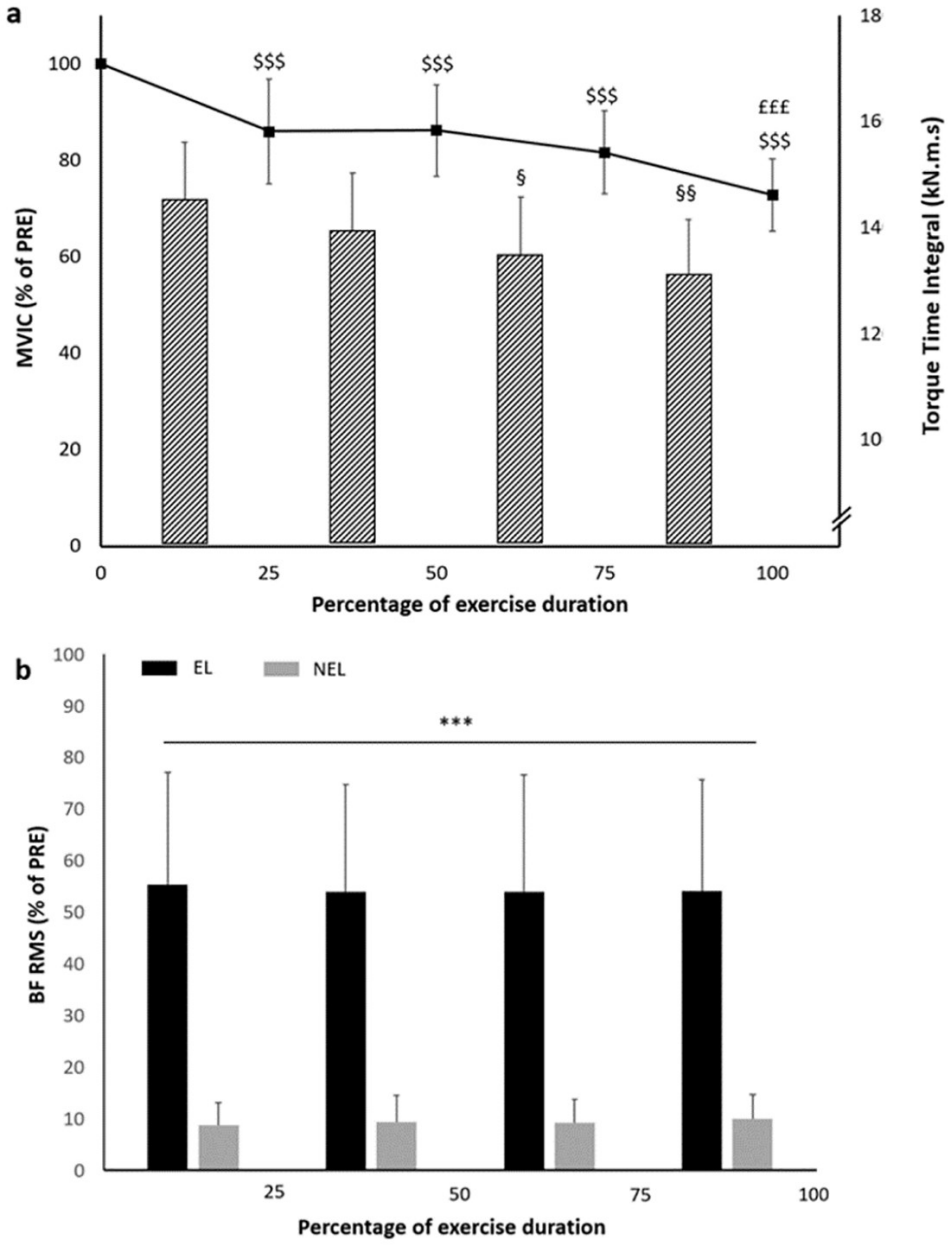
Measures analyzed during the unilateral submaximal eccentric exercise. Panel a: MVIC expressed as a percentage of the initial value (black line) and torque-time integral (dashed bars; mean ± standard error) of the exercised limb. Panel b: Surface electromyography of the biceps femoris muscle (BF RMS) expressed as a percentage of the initial value of both the EL (exercised limb; black bars) and the NEL (non-exercised limb; gray bars). ^$$$^ MVIC significantly different from initial value at p < 0.001. ^£££^ MVIC significantly different from initial, 25%, 50% and 75% values at p < 0.001. ^§^ and ^§§^ Torque-time integral produced during the third and the fourth quarter of exercise duration significantly different from the first quarter at p < 0.05 and p < 0.01, respectively. *** Pooled BF RMS values of the EL significantly different from the NEL at p < 0.001. MVIC Maximal voluntary isometric contraction.

Throughout the fatiguing exercise, a significant reduction of both the MVIC (F_(4,80)_ = 43.57; p < 0.001) and the torque-time integral (F_(3,64)_ = 4.93; p = 0.004) was observed. Lower MVIC values, expressed as a percentage of the initial value, were produced at 25%, 50%, 75% and at the end of the fatiguing exercise duration compared to PRE values (p < 0.001; Fig 2a). The MVIC value produced at the end of the fatiguing exercise was also lower than the values produced at 25%, 50% and 75% of exercise duration (p < 0.001; Fig 2a). The amount of torque-time integral achieved during the third and the fourth quarter of the fatiguing exercise duration was smaller than the one produced during the first quarter (p = 0.042 and p = 0.003, respectively; Fig 2a). A significant main effect of the limb was noted for the BF RMS values during the fatiguing exercise (F_(1,128)_ = 525.94; p < 0.001; Fig 2b) with pooled mean values of the EL (54.35 ± 21.67%) greater than those of the NEL (9.33 ± 4.74%) regardless of the fatiguing exercise duration (p < 0.001).

### PRE, POST and POST24 measurements

#### MVICs

A significant limb × time interaction was observed for MVIC (F_(2,96)_ = 6.19; p = 0.003). A significant reduction in MVIC was observed between PRE and POST in the EL (-28.09 ± 6.47%; p < 0.001; ES = 1.19; Fig 4a) and in the NEL (-8.52 ± 16.16%; p = 0.032; ES = 0.56; Fig 4a). No significant correlation was found between the MVIC reduction in the EL and the MVIC reduction in the NEL (r = 0.16; p > 0.05). MVIC torques measured at POST24 were no longer different from PRE values for both limbs (p > 0.05).

#### Central and peripheral fatigue indicators

**Fig 3:**
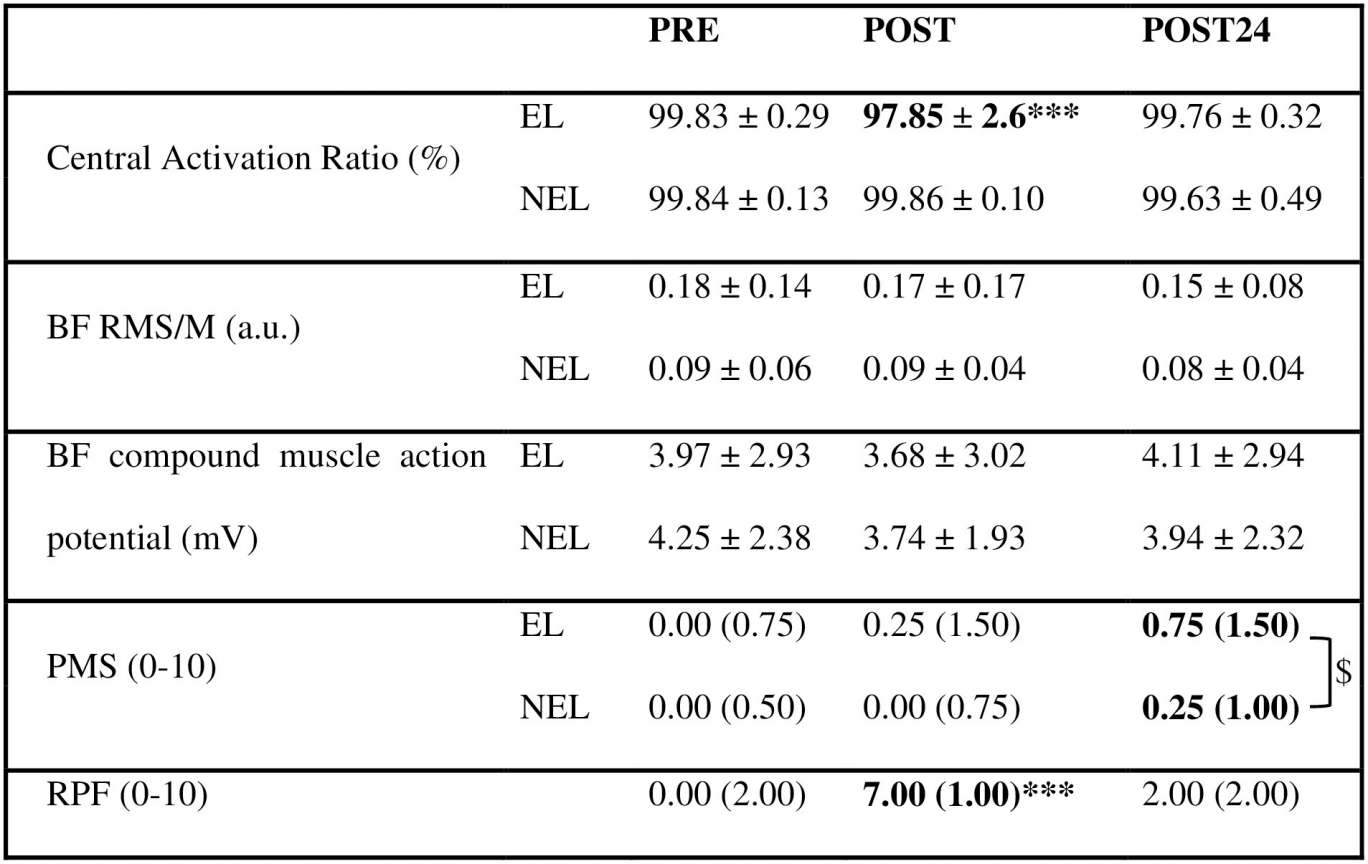
Effects of the unilateral submaximal eccentric exercise on knee flexor’s of neuromuscular function and perceived muscle soreness (PMS) of both the EL (exercised limb) and the NEL (non-exercised limb) as well as global perceived fatigue (RPF). Values in bold are statistically significant. *** Significantly different from PRE values at p < 0.001. ^$^ PMS scores of both the EL and NEL significantly greater at POST24 compared to PRE at p < 0.05. PMS and RPF scores are presented as median (interquartile range). BF Biceps Femoris, BF RMS/M Root Mean Square values of BF muscle normalized to the respective BF compound muscle action potential.

**Fig 4:**
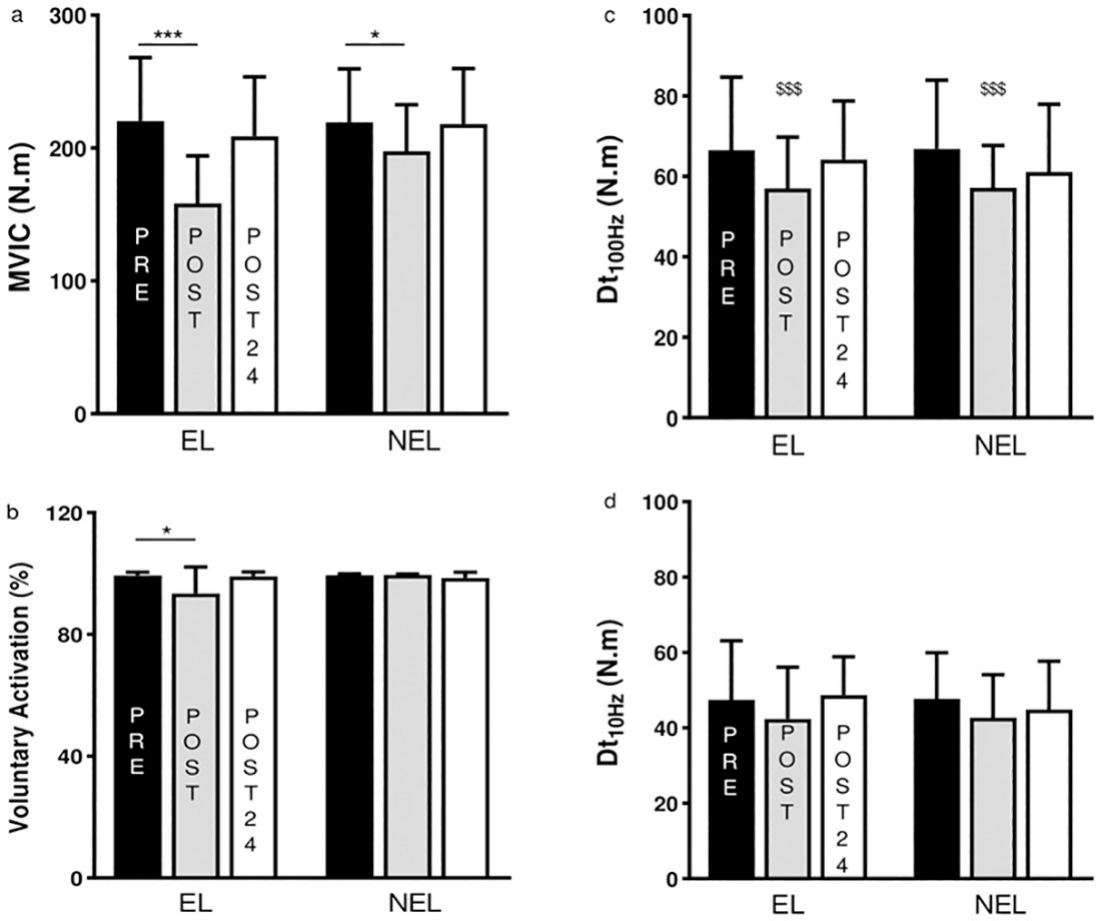
Effects of the unilateral submaximal eccentric exercise on knee flexor’s neuromuscular function. Panel a: MVIC (Maximal voluntary isometric contraction). Panel b: voluntary activation. Panel c: Dt_100Hz_ (Potentiated torque evoked at 100 Hz). Panel d: Dt_10Hz_ (Potentiated torque evoked at 10 Hz). Measures realized before (PRE; black bars), immediately after (POST; gray bars) and 24 (POST24; white bars) hours after the exercise in both the EL (exercised limb) and the NEL (non-exercised limb). * and *** indicate significant differences between PRE and POST at p < 0.05 and p < 0.001, respectively. ^$$$^ pooled values of both the EL and the NEL measured at POST significantly different from pooled EL and NEL values measured at PRE at p < 0.001.

A significant limb × time interaction was observed for both VA (F_(2,96)_ = 8.11; p = 0.001) and CAR values (F_(2,96)_ = 10.04; p < 0.001). A significant decrease in VA (-6.00 ± 8.24%; p < 0.001; ES = 0.87; Fig 4b) and CAR (-1.99 ± 2.48%; p < 0.001; ES = 0.93; Fig 3) was observed between PRE and POST in the EL, whereas no significant modification was noticed in the NEL. At POST, moderate significant correlations were observed in the EL between VA decrease and MVIC loss (r = 0.49, p = 0.048), as well as between CAR diminution and MVIC loss (r = 0.48, p = 0.049). At POST24, VA and CAR had returned to PRE values. No limb × time interaction was found in the BF RMS/M (F_(2,95)_ = 0.307; p = 0.737) and BF compound muscle action potential (F_(2,96)_ = 0.372; p = 0.691) values in both limbs (Fig 3).

While no limb × time interaction was noted, a significant time effect was observed for Dt_100Hz_ (F_(2,96)_ = 7.37; p = 0.001; Fig 4c) and a tendency was also noted for Dt_10Hz_ (F_(2,96)_ = 2.86; p = 0.06; Fig 4d). Regardless of the limb, Dt_100Hz_ decreased between PRE and POST (-11.56 ± 17.94%; p = 0.001; ES = 0.62). At POST, in the EL, the Dt_100Hz_ impairment was moderately correlated with the MVIC loss (r = 0.57, p = 0.018). POST24 values were not different from PRE values (p > 0.05). No interaction nor main effect was found (F_(2,96)_ = 0.593; p = 0.555) for the Dt_10Hz_-to-Dt_100Hz_ ratio. No significant correlation was evidenced between Dt_100Hz_ alterations and MVIC reduction measured in the NEL at POST (r = 0.31; p > 0.05). In addition, no correlation was observed between the immediate peripheral alterations of the EL and NEL (r = 0.02; p > 0.05).

#### Perceived fatigue and muscle soreness scores

A significant time effect was found for RPF scores (F_(2,48)_ = 77.45; p < 0.001). RPF scores significantly increased at POST compared to PRE (p < 0.001; ES = 1.75; Fig 3). RPF scores measured at POST24 were no longer different from PRE values (p > 0.05). At POST, no significant correlation was noted for increased RPF scores and MVIC reductions observed in the EL (r = 0.001; p > 0.05) and NEL (r = 0.20; p > 0.05). In addition, no significant correlation was found between the immediate increase in RPF scores and Dt_100Hz_ alterations reported in both the EL (r = 0.02; p > 0.05) and NEL (r = 0.40; p > 0.05).

Regardless of the limb, a significant time effect was noted for PMS scores (F_(2,96)_ = 3.95; p = 0.022; Fig 3) with greater scores at POST24 compared to PRE (p = 0.034; ES = 0.57).

## Discussion

This is the first study that highlights the occurrence of crossover fatigue on the knee flexors. With an exercise-induced MVIC reduction of -28% in the EL, the main findings of this study observed immediately after the fatiguing exercise (POST) were: i) a MVIC decrease of -8.5% in the contralateral NEL, ii) the MVIC reduction in the EL was related to central and peripheral alterations, iii) only an impaired contractile function was noted in the NEL, and iv) global perceived fatigue was increased. Significant greater perceived muscle soreness values were also observed 24 hours after the exercise while all measured variables had returned to baseline values. In addition, during the fatiguing exercise of the ipsilateral EL, involuntary electromyographic activity of the contralateral NEL did not exceed 10% of its maximal electromyographic activity.

To date, no study has investigated the effects of a unilateral exercise on the performance fatigability of the knee flexors of the exercised limb and its influence on performance fatigability of the contralateral homologous muscles. The immediate 28% MVIC decrease of the EL observed in the current investigation after an eccentric exercise is in line with the previous studies performed on other muscle groups showing MVIC impairment ranging from 16 to 50% [8, 9, 13]. Eccentric exercise is known to impair muscle contractile function, generally assessed through the measure of electrically evoked torques, immediately and/or during many days after the exercise [9, 17, 18]. Accordingly, we observed an immediate (i.e., between PRE and POST) reduction of Dt_100Hz_ (-12%) in the EL with no modification of the Dt_10Hz_-to-Dt_100Hz_ ratio. Since the BF compound muscle action potential values were unaffected by the eccentric exercise (i.e., sarcolemma excitability unchanged), the reduction of electrically evoked torques was a possible consequence of excitation-contraction coupling failure including increased intramuscular metabolites concentrations leading to impaired calcium release and/or decreased calcium sensitivity [37, 38]. Here, contrary to a previous study performed on the knee flexors [39], no modification of perceived muscle soreness was observed immediately after our unilateral eccentric exercise. This contradictory observation might be ascribable to the smaller amount of MVIC decrease observed here in comparison with Paschalis et al.’s [39] study (i.e., -28% vs -45%). Consequently, and considering the correlation between MVIC reduction and Dt_100Hz_ impairment, the knee flexors performance fatigability in the EL is due to an impaired muscle contractile function involving excitation-contraction coupling failure.

In addition to the above peripheral alterations, the presence of immediate central alterations in the EL was underlined by significant decreases in knee flexors’ VA (-6%; large ES = 0.87) and CAR (-2%; large ES = 0.93). Moreover, these central alterations (i.e., VA and CAR decreases) were significantly correlated with MVIC decreases in the EL. Previous experiments [27, 28] report non-significant changes ranging from 1.6 to 4.5% of the knee flexors’ voluntary activation after ecologically induced fatigue (i.e., simulated soccer match and repeated sprints). Conflicting results were also reported after intermittent isometric contractions of the knee flexors with a study failing to evidence voluntary activation impairment [23] whereas another showing a 8.5% reduction in voluntary activation [22]. To date, like the present investigation, only one recent study investigating crossover fatigue performed on the plantar flexor muscles [9] reported a significant decrease of voluntary activation (i.e., ~7%) immediately after a unilateral submaximal eccentric exercise. However, in contrast to our investigation, these authors did not observe central activation ratio modification. Although the muscle group assessed could partly account for these conflicting observations, the measures of voluntary activation and central activation ratio do not allow dissociating spinal from supraspinal changes [2, 40] implying that central alterations observed here may have occurred at both levels. The possible rise in intramuscular metabolites concentration associated with fatiguing exercises is known to increase the inhibitory actions from groups III-IV afferents which are highly activated during eccentric contractions [41, 42]. Groups III and IV afferents can modulate the descending central drive at both spinal (i.e., H-reflex studies) [43–45] and supraspinal level (i.e., corticospinal excitability studies) [19, 41, 45] after eccentric exercises on other muscle groups. However, it is extremely challenging to ascertain the exact origin of this modulation due the conflicting results reported in these studies. Central alterations were also assessed through the changes in the electromyographic activity (i.e., RMS/M) of the BF muscle, but no change was observed. However, only BF muscle activity was measured, which does not represent the entire descending central drive sent to the hamstrings. Along with central alterations, another interesting and original result of the present study that can be noted is the significant augmented RPF scores observed immediately after the exercise. A recent study also reported similar observations on the knee extensors after a submaximal fatiguing eccentric exercise [18]. Then, we can put forward the hypothesis that our submaximal eccentric exercise of the knee flexors is likely to induce to a global feeling of fatigue. To summarize, we originally observed that a unilateral submaximal eccentric exercise induced an immediate impaired performance fatigability of the EL knee flexors (including peripheral and central alterations) and an increase in global perceived fatigue. Although no significant correlation was observed between performance fatigability of the EL and perceived fatigue, our results may account for the possible interaction between performance fatigability and perceived fatigability [46].

Along with the immediate 28% MVIC decrease observed on the EL, a significant 8.5% MVIC drop was reported in the contralateral NEL (medium ES of 0.56), evidencing crossover fatigue in the knee flexors for the first time. Our result agrees with MVIC impairments of -13.7% and -7% reported after a unilateral eccentric exercise in the contralateral knee extensors and plantar flexors, respectively [8, 9]. The involuntary increases of electromyographic activity and force production reported in the non-exercised contralateral homologous muscle during maximal or fatiguing unilateral contractions of the ipsilateral muscle [3, 4] could partly contribute to crossover fatigue. Although observed in the first dorsal interosseous muscle, progressive increases of the electromyographic activity up to 38% of the maximal activity were reported with a crossover effect of ~10% [4]. Here though, the BF electromyographic activity of the NEL remained stable throughout the fatiguing exercise and corresponded to ~9% of its maximal electromyographic activity recorded during MVIC at PRE. Consequently, this level of activity is highly unlikely to have contributed to the MVIC reduction of the NEL.

Nevertheless, and to the same extent as in the EL, the Dt_100Hz_ (-12%) in the NEL was reduced immediately after the exercise. To the best of our knowledge, impaired electrically evoked torque in the contralateral homologous muscle (~ -20%) was only reported once in the literature after unilateral isometric exercises of the first dorsal interosseous muscle [4]. In addition, in a study investigating “non-local muscle fatigue”, impaired potentiated resting twitch of the elbow flexor muscles was also observed after one hour of downhill running [47]. In contrast, even if Marathamuthu et al. [9], recently observed a crossover fatigue in the non-exercised muscle group after a submaximal eccentric exercise, the resting twitch did not change. Due to the heterogeneity of the experimental designs between these studies and the present investigation, it is very challenging to explain such discrepancies. Nevertheless, muscle groups studied and/or methodological (i.e., single non-potentiated resting twitch vs. double potentiated twitch) differences could account for these divergent observations. A first explanation for the electrically evoked torque alterations of the present study could be related to an altered spinal excitability due to presynaptic inhibition of Ia afferent fibers through group III and IV afferents [43]. As mentioned above, considering that groups III-IV afferents are highly activated during eccentric contractions [41, 42] and modulate central drive through spinal projections [48], an impaired spinal excitability could have occurred. However, to the best of our knowledge, no study has yet investigated the influence of a unilateral eccentric exercise on the H-reflex response of the contralateral non-exercised muscle. Second, the metabolites accumulation induced by the exercise performed in the EL may spread to the NEL via the cardiovascular system. Although speculative here, such observations were evidenced in lower limbs following an intense upper limb muscles exercise [49, 50]. Third, in an animal study, the expression of heat shock proteins increased in the homologous contralateral muscle but also in other remote resting muscles immediately following a fatiguing exercise of the ipsilateral muscle induced by electrical stimulation [51]. Thanks to their experimental design, these authors concluded that both the sympathetic system and the neural system (e.g., through group III and IV afferents activation regulating the circulatory response during fatiguing muscle exercise [52] contributed to their observations. Taken together, it may be assumed that our submaximal eccentric exercise of the knee flexors led to a systemic adaptation possibly involving cardiovascular and neural systems. However, future studies should clearly investigate the occurrence of these systemic adaptations in human contralateral homologous muscles. In this regard, a recent study has suggested that a systemic adaptation could explain eccentric-induced crossover fatigue [9]. In this study, greater muscle soreness was reported immediately after the exercise and the authors argued that pain perception contributed to their significant 3.5% reduction of voluntary activation observed on the non-exercised contralateral muscles. As opposed to that, in our study, the PMS scores, voluntary activation and central activation ratio in the NEL remained unchanged immediately after the exercise (i.e., at POST). However, and although the significant increases in RPF scores were not correlated to MVIC decrease in the NEL, it cannot be ruled out that a general feeling of fatigue can contribute to a global systemic adaptation accounting for the immediate crossover fatigue reported in our investigation. Therefore, the peripheral alterations observed in our NEL knee flexors and the greater global perceived fatigue reinforce the possible influence of a cumulative adaptation [5] after the submaximal eccentric exercise performed here.

Recent crossover fatigue studies involving eccentric exercise have shown force impairment in the contralateral muscles that can last up to 48 hours [8, 9]. We therefore assessed both EL and NEL 24 hours after the exercise (POST24), but performance fatigability of the knee flexors in both limbs and global perceived fatigue had been fully recovered. Then, our observations conflict with these previous studies showing prolonged performance fatigability in the non-exercised contralateral muscles. Although the muscle groups tested and the fatiguing exercise differed from the present investigation, these two studies reported significant muscle pain up to 48 hours [8] and even 72 hours [9] in the contralateral limbs after their eccentric exercise, which could account for the performance fatigability observed in their non-exercised muscle groups. Here, performance fatigability of the knee flexors in both limbs was not significantly impaired anymore at POST24, despite the likely presence of muscle soreness indicated by the increase in PMS scores. Consequently, considering that the performance fatigability of the non-exercised plantar flexor muscles reported by Marathamuthu et al. [9] was similar (i.e., -7%) to the present investigation (i.e., -8.5%), it might be suggested that the muscle group assessed could partly explain these discrepancies. Future studies should ascertain the long lasting effects of an identical eccentric exercise on crossover fatigue performed on different muscle groups.

Although the present study evidenced interesting results concerning crossover fatigue in the non-exercised contralateral knee flexors, it is noteworthy to underline some limitations. First, the familiarization session including multiple isometric and eccentric contractions may have attenuated the possible occurrence of muscle damage [53, 54]. Second, the range of motion chosen during our unilateral submaximal eccentric exercise was not realized until full knee extension. Then, the eccentric contractions were not performed at long muscle length, possibly minimizing the incidence of muscle damage [55]. Third, the methodological approach used in the present investigation did not allow for the identification of the exact mechanism explaining the peripheral alterations observed in the NEL. Finally, considering that only male participants were included in this investigation, the generalization of our results to female participants should be tested in future studies.

## Conclusion

Unilateral submaximal eccentric hamstring exercise undeniably induced immediate crossover fatigue. The decrease in force production observed in both limbs has a different etiology. On the one hand, central activation failure and peripheral alterations (i.e., performance fatigability) can account for the exercise-induced fatigue in the EL but an increased global perceived fatigue (i.e., perceptions of fatigue) was also evidenced. On the other hand, the crossover fatigue observed in the NEL may be ascribable to a more complex interaction involving peripheral alterations and possibly a contribution of the increased global perceived fatigue observed. Interestingly, we observed that electrically evoked torques were impaired similarly in both limbs, but the exact mechanism accounting for this result in the NEL has still to be further investigated. Finally, a compensatory mechanism involving an involuntary increase of the electromyographic activity in the NEL does not seem to contribute to the crossover fatigue.

## References

[1] Kluger BM, Krupp LB, Enoka RM. Fatigue and fatigability in neurologic illnesses: proposal for a unified taxonomy. Neurology. 2013; 80(4): 409–16. doi: 10.1212/WNL.0b013e31827f07be 23339207 PMC3589241

[2] Gandevia SC. Spinal and supraspinal factors in human muscle fatigue. Physiological Reviews. 2001; 81(4): 1725–89. doi: 10.1152/physrev.2001.81.4.1725 11581501

[3] Zijdewind I, Kernell D. Bilateral interactions during contractions of intrinsic hand muscles. Journal of Neurophysioly. 2001; 85(5): 1907–13. doi: 10.1152/jn.2001.85.5.1907 11353007

[4] Post M, Bayrak S, Kernell D, Zijdewind I. Contralateral muscle activity and fatigue in the human first dorsal interosseous muscle. Journal of Applied Physiology. 2008; 105(1): 70–82. doi: 10.1152/japplphysiol.01298.2007 18450978

[5] Behm DG, Alizadeh S, Hadjizedah Anvar S, Hanlon C, Ramsay E, Mahmoud MMI, et al. Non-local muscle fatigue effects on muscle strength, power, and endurance in healthy individuals: a systematic review with meta-analysis. Sports Medicine. 2021; 51(9): 1893–1907. doi: 10.1007/s40279-021-01456-3 33818751

[6] Zijdewind I, Zwarts MJ, Kernell D. Influence of a voluntary fatigue test on the contralateral homologous muscle in humans? Neuroscience Letters. 1998; 253(1): 41–4. doi: 10.1016/s0304-3940(98)00609-0 9754800

[7] Doix ACM, Lefèvre F, Colson SS. Time course of the cross-over effect of fatigue on the contralateral muscle after unilateral exercise. PLoS One. 2013; 8(5): e64910. doi: 10.1371/journal.pone.0064910 23741417 PMC3669025

[8] Hedayatpour N, Izanloo Z, Falla D. The effect of eccentric exercise and delayed onset muscle soreness on the homologous muscle of the contralateral limb. Journal of Electromyography and Kinesiology. 2018; 41: 154–9. doi: 10.1016/j.jelekin.2018.06.003 29902705

[9] Marathamuthu S, Selvanayagam VS, Yusof A. Contralateral effects of eccentric exercise and DOMS of the plantar flexors: evidence of central involvement. Research Quaterly for Exercise and Sport. 2022; 93(2): 240–9. doi: 10.1080/02701367.2020.1819526 32976088

[10] Martin PG, Rattey J. Central fatigue explains sex differences in muscle fatigue and contralateral cross-over effects of maximal contractions. Pflügers Archiv. 2007; 454(6): 957–69. doi: 10.1007/s00424-007-0243-1 17342531

[11] Halperin I, Copithorne D, Behm DG. Unilateral isometric muscle fatigue decreases force production and activation of contralateral knee extensors but not elbow flexors. Applied Physiology, Nutrition and Metabolism. 2014; 39(12): 1338–44. 10.1139/apnm-2014-0109 25291403

[12] Kennedy DS, Fitzpatrick SC, Gandevia SC, Taylor JL. Fatigue-related firing of muscle nociceptors reduces voluntary activation of ipsilateral but not contralateral lower limb muscles. Journal of Applied Physiology. 2015; 118(4): 408–18. doi: 10.1152/japplphysiol.00375.2014 25525208

[13] Miller W, Jeon S, Ye X. An examination of acute cross-over effects following unilateral low intensity concentric and eccentric exercise. Sports Medicine and Health Science. 2020; 2(3):141–52. doi: 10.1016/j.smhs.2020.08.002 35782286 PMC9219316

[14] Rattey J, Martin PG, Kay D, Cannon J, Marino FE. Contralateral muscle fatigue in human quadriceps muscle: evidence for a centrally mediated fatigue response and cross-over effect. Pflügers Archiv. 2006; 452(2): 199–207. doi: 10.1007/s00424-005-0027-4 16365782

[15] Doix ACM, Wachholz F, Marterer N, Immler L, Insam K, Federolf PA. Is the cross-over effect of a unilateral high-intensity leg extension influenced by the sex of the participants? Biology of Sex Differences. 2018; 9(1): 29. doi: 10.1186/s13293-018-0188-4 29954447 PMC6022493

[16] Grabiner MD, Owings TM. Effects of eccentrically and concentrically induced unilateral fatigue on the involved and uninvolved limbs. Journal of Electromyography and Kinesiology. 1999; 9(3): 185–89. doi: 10.1016/s1050-6411(98)00031-5 10328413

[17] Skurvydas A, Brazaitis M, Venckunas T, Kamandulis S, Stanislovaitis A, Zuoza A. The effect of sports specialization on musculus quadriceps function after exercise-induced muscle damage. Applied Physiology, Nutrition and Metabolism. 2011; 36(6):873–80. 10.1139/h11-112 22050132

[18] Da Silva F, Monjo F, Zghal F, Chorin F, Guérin O, Colson SS. Altered position sense after submaximal eccentric exercise-inducing central fatigue. Medicine and Science in Sports and Exercise. 2021; 53(1): 218–27. doi: 10.1249/MSS.0000000000002444 32694369

[19] Garnier YM, Paizis C, Lepers R. Corticospinal changes induced by fatiguing eccentric versus concentric exercise. European Journal of Sport Science. 2019; 19(2):166–76. doi: 10.1080/17461391.2018.1497090 30016203

[20] Halperin I, Chapman DW, Behm DG. Non-local muscle fatigue: effects and possible mechanisms. European Journal of Applied Physiology. 2015; 115(10): 2031–48. doi: 10.1007/s00421-015-3249-y 26330274

[21] Miller W, Kang M, Jeon S, Ye X. A meta-analysis of non-local heterologous Muscle Fatigue. Journal of Trainology. 2019;8(1): 9–18.

[22] Coratella G, Grosprêtre S, Gimenez P, Mourot L. Greater fatigability in knee-flexors vs. knee-extensors after a standardized fatiguing protocol. European Journal of Sport Science. 2018; 18(8): 1110–18. doi: 10.1080/17461391.2018.1469674 29738677

[23] Massamba A, Hucteau E, Mallard J, Ducrocq GP, Favret F, Hureau TJ. Exercise-induced fatigue in hamstring versus quadriceps muscles and consequences on the torque-duration relationship in men. Medicine and Science in Sports and Exercise. 2022; 54(12): 2099–2108. doi: 10.1249/MSS.0000000000003007 35868018

[24] Askling CM, Tengvar M, Thorstensson A. Acute hamstring injuries in Swedish elite football: A prospective randomised controlled clinical trial comparing two rehabilitation protocols. British Journal of Sports Medicine. 2013; 47(15): 953–9. doi: 10.1136/bjsports-2013-092165 23536466

[25] Corcelle B, Morin JB, Gerus P, Giacomo JP, Piponnier E. New field ergometer to reproducibly measure maximum strength and rate of force development of hamstrings. Science & Sports. 2022; 37(8): 802.e1–802.e8.

[26] Hermens HJ, Freriks B, Disselhorst-Klug C, Rau G. Development of recommendations for SEMG sensors and sensor placement procedures. Journal of Electromyography and Kinesiology. 2000; 10(5): 361–74. doi: 10.1016/s1050-6411(00)00027-4 11018445

[27] Marshall PWM, Lovell R, Jeppesen GK, Andersen K, Siegler JC. Hamstring muscle fatigue and central motor output during a simulated soccer match. PLoS One. 2014; 9(7): e102753 doi: 10.1371/journal.pone.0102753 25047547 PMC4105441

[28] Baumert P, Temple S, Stanley JM, Cocks M, Strauss JA, Shepherd SO, et al. Neuromuscular fatigue and recovery after strenuous exercise depends on skeletal muscle size and stem cell characteristics. Scientific Reports. 2021; 11(1): 7733. doi: 10.1038/s41598-021-87195-x 33833326 PMC8032692

[29] Micklewright D, St Clair Gibson A, Gladwell V, al Salman A. Development and validity of the rating-of-fatigue scale. Sports Medicine. 2017; 47(11): 2375–93. doi: 10.1007/s40279-017-0711-5 28283993 PMC5633636

[30] Brownstein CG, Rimaud D, Singh B, Fruleux-Santos LA, Sorg M, Micklewright D, et al. French translation and validation of the rating-of-fatigue scale. Sports Med Open. 2021; 7(1): 25. doi: 10.1186/s40798-021-00316-8 33829336 PMC8026791

[31] Proske U, Gregory JE, Morgan DL, Percival P, Weerakkody NS, Canny BJ. Force matching errors following eccentric exercise. Human Movement Science. 2004; 23(3–4): 365–78. doi: 10.1016/j.humov.2004.08.012 15541523

[32] Edwards RH, Hill DK, Jones DA, Merton PA. Fatigue of long duration in human skeletal muscle after exercise. Journal of Physiology. 1977; 272(3): 769–78. doi: 10.1113/jphysiol.1977.sp012072 592214 PMC1353654

[33] Millet GY, Martin V, Martin A, Vergès S. Electrical stimulation for testing neuromuscular function: from sport to pathology. European Journal of Applied Physiology. 2011; 111(10): 2489–500. doi: 10.1007/s00421-011-1996-y 21590274

[34] Place N, Millet GY. Quantification of neuromuscular fatigue: what do we do wrong and why? Sports Medicine. 2020; 50(3): 439–47. doi: 10.1007/s40279-019-01203-9 31713783

[35] Barr DJ, Levy R, Scheepers C, Tily HJ. Random effects structure for confirmatory hypothesis testing: Keep it maximal. Journal of Memory and Language. 2013, 68(3): 255–78. doi: 10.1016/j.jml.2012.11.001 24403724 PMC3881361

[36] Schober P, Boer C, Schwarte LA. Correlation coefficients: appropriate use and interpretation. Anesthesia and Analgesia. 2018; 126(5): 1763–68. doi: 10.1213/ANE.0000000000002864 29481436

[37] Allen DG, Lamb GD, Westerblad H. Skeletal muscle fatigue: cellular mechanisms. Physiological reviews. 2008; 88(1): 287–332. doi: 10.1152/physrev.00015.2007 18195089

[38] Place N, Yamada T, Bruton JD, Westerblad H. Muscle fatigue: from observations in humans to underlying mechanisms studied in intact single muscle fibres. European Journal of Applied Physiology. 2010; 110(1): 1–15. doi: 10.1007/s00421-010-1480-0 20419312

[39] Paschalis V, Nikolaidis MG, Giakas G, Jamurtas AZ, Owolabi EO, Koutedakis Y. Position sense and reaction angle after eccentric exercise: The repeated bout effect. European Journal of Applied Physiology. 2008; 103(1): 9–18. doi: 10.1007/s00421-007-0663-9 18172668

[40] Dotan R, Woods S, Contessa P. On the reliability and validity of central fatigue determination. European Journal of Applied Physiology. 2021; 121(9): 2393–411. doi: 10.1007/s00421-021-04700-w 33966110

[41] Löscher WN, Nordlund MM. Central fatigue and motor cortical excitability during repeated shortening and lengthening actions. Muscle Nerve. 2002; 25(6): 864–72. doi: 10.1002/mus.10124 12115976

[42] Martin V, Dousset E, Laurin J, Gondin J, Gautier M, Decherchi P. Group III and IV muscle afferent discharge patterns after repeated lengthening and shortening actions. Muscle Nerve. 2009; 40(5): 827–37. doi: 10.1002/mus.21368 19626674

[43] Vangsgaard S, Nørgaard LT, Flaskager BK, Søgaard K, Taylor JL, Madeleine P. Eccentric exercise inhibits the H reflex in the middle part of the trapezius muscle. European Journal of Applied Physiology. 2013; 113(1): 77–87. doi: 10.1007/s00421-012-2412-y 22573465

[44] Oza PD, Dudley-Javoroski S, Shields RK. Dynamic fatigue does not alter soleus H-reflexes conditioned by homonymous or heteronymous pathways. Motor Control. 2017; 21(3): 345–58. doi: 10.1123/mc.2016-0030 27736308 PMC5604332

[45] Škarabot J, Ansdell XP, Temesi J, Howatson G, Goodall S, Durbaba R. Neurophysiological responses and adaptation following repeated bouts of maximal lengthening contractions in young and older adults. Journal of Applied Physiology. 2019; 127(5): 1224–37. doi: 10.1152/japplphysiol.00494.2019 31513444

[46] Enoka RM, Duchateau J. Translating fatigue to human performance. Medicine and Science in Sports and Exercise. 2016; 48(11): 2228–38. doi: 10.1249/MSS.0000000000000929 27015386 PMC5035715

[47] Ye X, Benton RJ, Miller WM, Jeon S, Song JS. Downhill running impairs peripheral but not central neuromuscular indices in elbow flexor muscles. Sports Medicine and Health Science. 2021; 3(2): 101–9. doi: 10.1016/j.smhs.2021.03.001 35782164 PMC9219267

[48] Degtyarenko AM, Kaufman MP. Spinoreticular neurons that receive group III input are inhibited by MLR stimulation. Journal of Applied Physiology. 2002; 93(1): 92–98. doi: 10.1152/japplphysiol.00072.2002 12070191

[49] Bangsbo J, Madsen K, Kiens B, Richter EA. Effect of muscle acidity on muscle metabolism and fatigue during intense exercise in man. Journal of Physiology. 1996; 495(2): 587–96. doi: 10.1113/jphysiol.1996.sp021618 8887768 PMC1160816

[50] Johnson MA, Mills DE, Brown PI, Sharpe GR. Prior upper body exercise reduces cycling work capacity but not critical power. Medicine and Science in Sports and Exercise. 2014; 46(4): 802–8. doi: 10.1249/MSS.0000000000000159 24042306

[51] Jammes Y, Steinberg JG, By Y, Brerro-Saby C, Condo J, Olivier M, et al. Fatiguing stimulation of one skeletal muscle triggers heat shock protein activation in several rat organs: The role of muscle innervation. Journal of Experimental Biology. 2012; 215(22): 4041–8. doi: 10.1242/jeb.074427 22899526

[52] Kaufman MP, Hayes SG. The exercise pressor reflex. Clinical Autonomic Research. 2002; 12(6): 429–39. doi: 10.1007/s10286-002-0059-1 12598947

[53] Nosaka K, Newton M, Sacco P. Delayed-onset muscle soreness does not reflect the magnitude of eccentric exercise-induced muscle damage. Scandinavian Journal of Medicine and Science in Sports. 2002; 12(6): 337–46. doi: 10.1034/j.1600-0838.2002.10178.x 12453160

[54] Hyldahl RD, Chen TC, Nosaka K. Mechanisms and mediators of the skeletal muscle repeated bout effect. Exercise and Sport Science Review. 2017; 45(1): 24–33. doi: 10.1249/JES.0000000000000095 27782911

[55] Nosaka K, Sakamoto K. Effect of elbow joint angle on the magnitude of muscle damage to the elbow flexors. Medicine and Science in Sports and Exercise. 2001; 33(1): 22–9. doi: 10.1097/00005768-200101000-00005 11194107

